# A cross-sectional study to estimate prevalence of periodontal disease in a population of dogs (*Canis familiaris*) in commercial breeding facilities in Indiana and Illinois

**DOI:** 10.1371/journal.pone.0191395

**Published:** 2018-01-18

**Authors:** Judith L. Stella, Amy E. Bauer, Candace C. Croney

**Affiliations:** 1 USDA-APHIS, Purdue University, W. Lafayette, Indiana, United States of America; 2 Departement of Comparative Pathobiology, Purdue University, W. Lafayette, Indiana, United States of America; University of Bari, ITALY

## Abstract

The objectives of this cross-sectional study were: 1) to estimate the prevalence and characterize the severity of periodontal disease in a population of dogs housed in commercial breeding facilities; 2) to characterize PD preventive care utilized by facility owners; and 3) to assess inter-rater reliability of a visual scoring assessment tool. Adult dogs (N = 445) representing 42 breeds at 24 CB facilities in Indiana and Illinois were assessed. Periodontal disease was scored visually using the American Veterinary Dental Collage 0-IV scale. Inter-rater reliability was assessed on 198 dogs and facility owners were asked to provide information about the preventive care utilized. The overall prevalence of periodontal disease (Grades I-IV) was 86.3% (95% CI: 82.9, 89.3). An ordered logistic regression analysis found age (OR = 1.4; 95% CI 1.24, 1.54; P<0.0001), facility (OR = 1.13; 95% CI 1.09, 1.18; P<0.0001), sex (OR = 1.7; 95% CI 1.12, 2.65; P = 0.013), and non-professional dental scaling (OR = 2.82; 95% CI 1.34, 5.91; P = 0.006) to be statistically significant. Inter-rater reliability analysis found agreement to be 86.2%, with a weighted kappa of 0.4731 (95% CI 0.3847, 0.5615) indicating moderate agreement. Risk of periodontal disease increased with increasing age. Additionally, a trend toward decreasing risk with increasing weight was also found, although it was not statistically significant. The trends identified agree with studies that have evaluated periodontal disease in the companion dog population and do not support the assumption that the dental health of dogs in commercial breeding facilities is worse than that of the population as a whole. Although there were few cases of severe periodontal disease and all facilities employed some type of preventive care in this sample, the large number of dogs with some degree of disease (Grades I-IV) suggests that further investigation of preventive care is warranted.

## Introduction

Periodontal disease (PD) is a significant veterinary health problem of companion dogs with the majority of dogs reported to be affected with some degree of PD by two years of age. Estimated prevalence ranges from as high as 80% [1], [2] in the US to a reported prevalence of 9.3% in England [3], and a prevalence of dental calculus of 31% in Belgium [4]. In a population of companion dogs in the Czech Republic 60.0% of dogs had periodontitis, 61.3% had calculus and 33.8% had missing teeth [5]. While prevalence estimates range across studies, PD consistently ranks among the top two or three disorders affecting companion dogs.

This disease manifests as a result of infection and inflammation of the gums, bone and other tissues surrounding and supporting the teeth [6]. Chronic PD is initiated by an overgrowth of predominantly Gram-negative, anaerobic bacteria in subgingival sites often leading to inflammation and possibly, systemic disease. Two stages of PD exist, gingivitis and periodontitis. The initial, reversible stage is gingivitis which is an inflammation of the gingiva [2]. Periodontitis, an inflammation of the supporting structures of the tooth, is the later, irreversible stage of the disease [2]. Prevalence of PD has been reported to significantly increase with age and decrease with increasing body weight (i.e. size of dog) [7], suggesting that smaller breed dogs may be at greater risk than larger breed dogs. Additionally, Logan (2006) in a review of dietary influences on PD, reported an association of gingivitis with nutritional deficiencies of vitamins A, C, D and E, and the B vitamins folic acid, niacin, pantothenic acid and riboflavin [8].

The gold standard of preventive care is daily brushing of the dog’s teeth but this is not always practical and caretaker compliance is typically low. For example, a phone survey of 51 dog guardians assessed compliance with recommended daily brushing six months following a veterinary dental procedure. After six months, 53% of the guardians reported brushing several times per week while 38% had discontinued brushing altogether [9]. Studies in laboratory beagles have shown that in order to be effective, brushing at minimum three times per week was necessary to maintain oral health, while daily brushing was needed in dogs with gingivitis [10], [11]. The second best option for preventive care is to feed approved dental diets (e.g. Hill’s t/d diet^,^ Colgate-Palmolive, Topeka, KS) and treats (e.g. CET enzymatic chews^,^ Virbac USA, Ft Worth, TX) [12]. While it is commonly believed that a dry kibble diet decreases risk of PD, this has not been proven [13]. Additionally, it is recommended that all dogs be evaluated at least annually by a veterinarian to assess the need for preventive dental care performed under anesthesia (professional dental cleaning).

Due to the costs associated with professional dental cleaning as well as the perceived and real risks of anesthesia, dog breeders and other caretakers may seek alternative methods of maintaining dental health. These include Anesthesia-Free Dentistry, termed Non-Professional Dental Scaling (NPDS) by the American Veterinary Dental College (AVDC) [14]. There are significant welfare concerns relative to non-professional scaling, including risk of injury, pain and discomfort to the dogs, improper and incomplete cleaning, and damage to the teeth. It is unknown how common this practice is in both the dog breeding community as well as among companion dog caretakers.

An additional concern with PD is its association with an increased risk of developing systemic disease resulting from the release of inflammatory cells and by-products in response to bacteremia. Systemic diseases where an association with PD has been documented include chronic bronchitis, pulmonary fibrosis, endocarditis, interstitial nephritis, glomerulonephritis, and hepatitis [15]. The most commonly cited secondary organ system affected by PD is the cardiovascular system. Cross-sectional, case-control, and longitudinal studies have shown this association in humans [16], while observational studies have pointed to the same association in dogs [15]. For example, in a historical observational cohort study of 59,296 dogs, a significant association was found between the severity of PD and the risk of cardiovascular-related disease conditions such as endocarditis and cardiomyopathy. The authors therefore concluded that greater awareness of the importance of canine dental health and routine preventive care would improve overall health [15]. The American Veterinary Dental Society cautions pet guardians “that oral bacteria will be filtered out by the kidney and liver, and can cause micro-abscesses within these organs. This leads to a decrease in function of these vital organs over time. In addition, it has been suggested that these bacteria can become attached to the heart valves and cause a disease called endocarditis” [15].

PD is often characterized as more prevalent, more severe and less likely to be treated by a veterinarian in dogs used for commercial breeding than in companion or hobby breeding dogs. Commercial breeding (CB) facilities are often portrayed as maintaining dogs in substandard conditions relative to housing, sanitation and veterinary care. One area emphasized as being of notable concern is PD. The Humane Society Veterinary Medical Association noted that “severe periodontal disease is routinely seen in breeding stock…Eight year old breeding stock rarely have many teeth left…Dental disease is likely a result of poor diet, poor air quality/ventilation, and poor grooming practices. The dogs often have a painful, infected mouth with loose and missing teeth”[17]. These issues, if validated, represent significant animal welfare problems. However, to date no scientific evaluation of the prevalence or severity of PD or the management practices employed to address PD has been conducted in CB facilities.

Of particular concern to dog breeders and animal health professionals is the health of the puppies produced. Recent studies suggest that PD, as a source of subclinical and persistent infection, may induce systemic inflammatory responses that increase the risk of adverse pregnancy outcomes. Systematic reviews of the literature in humans suggested an association between PD and premature low birth weight (PTLBW), miscarriage and pre-eclampsia [18], [19]. Hope et al. [20] recently reported an association between plaque coverage and women who were considered to be at risk of pre-term birth. The results indicated that early localized periodontitis of the patient during pregnancy can be regarded as an important risk factor for premature birth. Additionally, a case-control study of 85 women found an increased risk (OR 5.46) of premature birth and lower birth weight babies in women with PD compared to those without [21]. Anecdotally, dog breeders who have incorporated routine dental care into the management of their breeding stock have reported increased litter size and healthier puppies (personnel communication). No scientific evaluations of the association between PD and pregnancy outcomes have yet been reported in dogs.

While assumptions about the welfare of dogs in CB facilities are extensively publicized, research on their welfare is limited. Given the significant implications of dental health for the well-being of breeding dogs, the aims of this study were to: 1) estimate the prevalence and severity of PD in dogs maintained at CB facilities; 2) characterize the type of preventive dental care and veterinary dental care utilized by commercial dog breeders; and 3) assess the inter-rater reliability (IRR) of scoring PD using a visual assessment tool. In this paper, we report that all specific aims were accomplished.

## Materials and methods

### Facilities

Twenty four USDA licensed facilities in Indiana and Illinois that volunteered to participate were visited. All facilities were owned and managed by members of the Amish community.

### Raters

The raters (AB and JS) were both women. Rater 1 holds a DVM and has experience in small animal clinical practice, including general dentistry practice. Rater 2 holds a PhD in animal welfare science and has experience as a veterinary technician in small animal clinical practice, including general dentistry practice. One hundred and six dogs were evaluated by Rater 1 only, 141 dogs were evaluated by rater 2 only, and 198 dogs were evaluated by both raters.

### Subjects

Adult dogs over one year of age were examined. Bitches in the last two weeks of gestation, those nursing puppies or any dog that exhibited overt fear or distress prior to or during the oral examination were excluded from the study. Based on a reported 80% prevalence of PD in companion dogs (p), a desired 95% confidence level, and desired precision of 5% (d), a minimum sample size of 246 dogs was calculated in an open source statistical software program (OpenEpi, Version 3.01, www.OpenEpi.com) using the formula: Sample size n = [Np(1-p)]/[d^2^/Z^2^_1-a/2_*(N-1)+p*(1-p)]. N is the population size and for these calculations; an N of 1,000,000 was used to account for the large population of interest. As there is little non-anecdotal information about the prevalence of PD in dogs housed in CB facilities, a maximum sample size of 384 dogs was also calculated using the same formula, but replacing the value of p with 50%. Thirty dogs (large facilities) or all dogs (small facilities) meeting inclusion criteria were assessed at each site (n = 445).

### PD score

**Fig 1:**
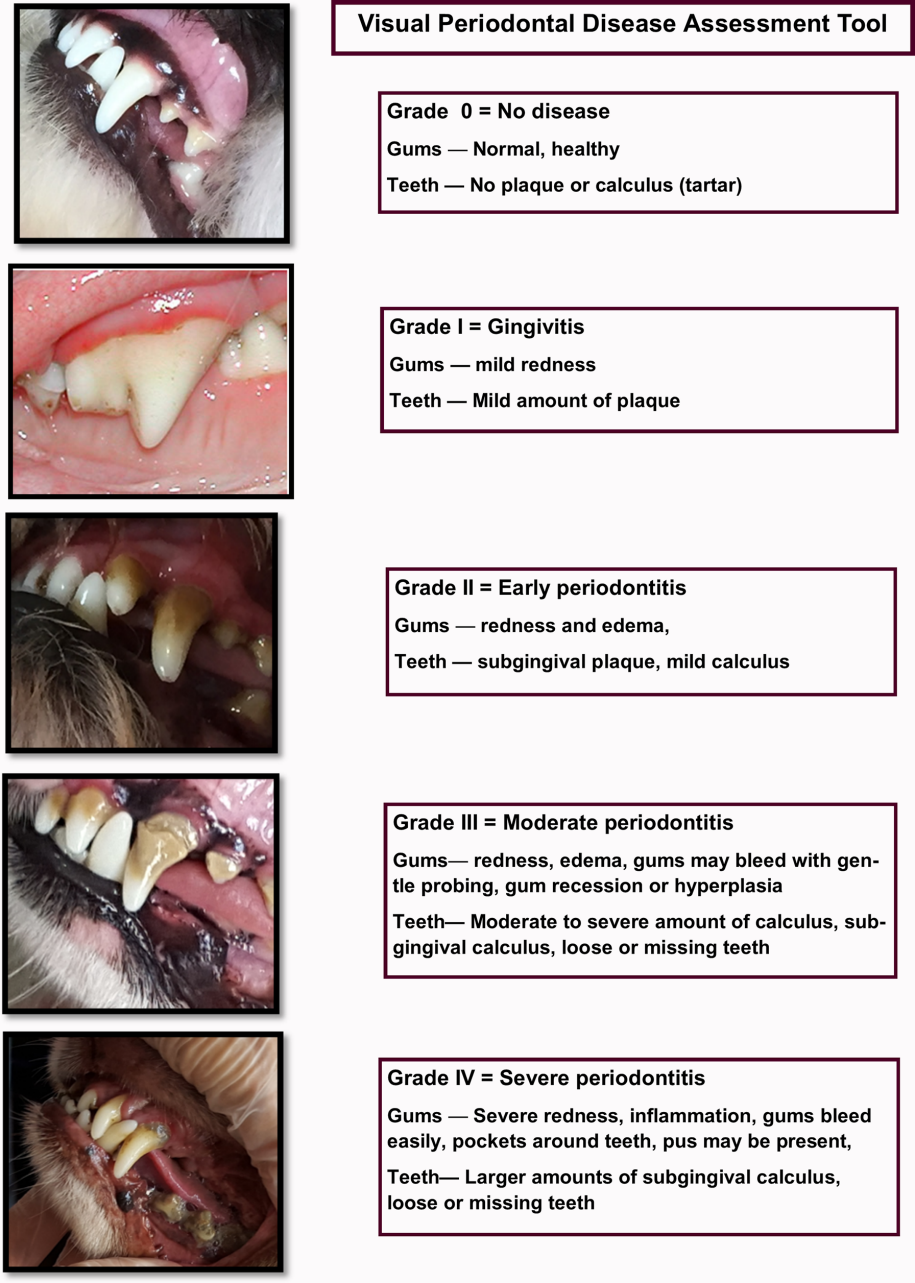
Visual assessment scale. The tool used to score severity of PD.

Each dog had its teeth and gums independently examined by one of the raters for gingivitis, dental calculus, and loose or missing teeth. A subset of dogs (n = 198) was examined by both raters to assess inter-rater reliability (IRR) of the assessment tool. The lips and cheeks were retracted to allow visualization of all teeth and gingiva. If the dog allowed, the mouth was also opened slightly to facilitate examination of the mandibular teeth and gingival margin. In order to minimize the stress of oral examine, the mouth was not opened widely enough to permit examination of the lingual and palatal aspects of the teeth. Although the presence of plaque or calculus was noted, the raters focused on the gingival margin to detect redness, edema, recession, and evidence of gingival bleeding. A full mouth score was recorded for each dog based on the tooth/teeth with the most severe PD. The tool used was based on the American Veterinary Dental College (AVDC) 0-IV scale but modified for use as a field ready, visual assessment scale (**Fig 1).** Grade 0 indicates a healthy mouth with no evidence of redness or swelling at the gingival margin of any teeth; dogs with Grade I have a tooth or teeth with redness at the gingival margin; Grade II indicates the presence of redness extending beyond the gingival margin as well as swelling indicative of edema; mouths staged as Grade III have evidence of bleeding, gingival recession or gingival hyperplasia in addition to redness and edema; finally, dogs with mouths staged as Grade IV have extensive gingival recession, evidence of infection in addition to inflammation, and loose or missing teeth. Upon completion of the oral examination, caretakers were informed of any abnormalities detected including PD, chipped, broken, worn, or missing teeth.

### Body condition score

Body condition of dogs was collected to determine its possible relationship to severity of PD. Body condition was scored using a 5 point scale (1 = emaciated, 2 = slightly underweight, 3 = ideal, 4 = slightly overweight, 5 = obese) (https://www.aaha.org/public_documents/professional/guidelines/weightmgmt_bodyconditionscoring.pdf).

### Skull morphology

Breeds were classified as brachycephalic, mesaticephalic, or dolichocephalic to assess the relationship between skull morphology and severity of PD. Breeds were classified based on a review of the American Kennel Club (AKC) breed standards. In this study, we included dogs in the brachycephalic group if the AKC breed standard described the muzzle as “short” and the muzzle to skull ratio as 1/3:2/3 or less [22].

### Management practices

Facility owners were asked to provide information about dental care including diet, provision of dental chews (e.g. enzymatic chews), bones or other mechanical cleaning devices, water additives (e.g. chlorhexidine), topical treatments (e.g. foams), frequency of veterinary dental examinations, and frequency of NPDS.

Informed consent was obtained from all facility owners who volunteered for the study. The Institutional Animal Care and Use Committee, Institutional Review Board, and the Clinical Trials Office in the College of Veterinary Medicine at Purdue University approved all experimental procedures.

### Statistical analysis

A prevalence estimate was calculated (including 95% confidence intervals) for the sample as whole as follows: (number of dogs with evidence of PD)/(total number of dogs) using an open source statistical software program (OpenEpi, Version 3.01, www.OpenEpi.com).

An ordered logistic regression model was used to analyze the data where PD severity was the outcome variable and independent variables included in the model were age (in years), facility, sex, breed, preventive care interventions, and skull morphology. Breed, skull morphology, and preventive care intervention were treated as factor variables so breed had four outcomes based on the average adult weight for that breed, >36.4 kg (>80 lb), 16.4–36.4 kg (36–80 lb), 5–16.3 kg (11–35.9 lb), <5 kg (< 11 lb), the skull morphology of each breed was characterized as either mesaticephalic, brachycephalic, or dolichocephalic, and preventive care intervention was addition of chlorhexidine to the water, provision of a chew item, NPDS, or more than one of the above. Analysis was completed using commercially available statistical software (StataCorp LP, College Station, TX, USA).

Inter-rater reliability of the visual scoring tool was assessed using a weighted kappa with the equation *1 − |i − j|/(k − 1)*, where a score of 1 indicates perfect agreement, a score of 0.6667 means that the raters are “one apart”, 0.3333 means that they are “two apart” and agreement is no greater than that due to chance when the score is zero [22] using commercially available statistical software (StataCorp LP, College Station, TX, USA). Graphical representation was performed using graphical software (GraphPad Software Inc., La Jolla, CA, USA).

## Results

A total of 445 dogs (F = 342, M = 103) at 24 facilities were assessed. Mean age was 3.15 years and ranged from 1–11.9 years (**Table 1)**. Forty two breeds, ranging in size from toy to giant breeds, were included in the study population (**Table 1)**.

**Table 1:** Demographic information of the study population by facility. N = number of dogs examined, F = females, M = males; mean age in years, SD = standard deviation.

Facility	N (F/M)	Mean Age (+/-SD)	Breeds
1	30 (25/5)	3.06 (1.55)	Maltese, Min. Pinscher, Yorkshire terrier
2	23 (17/6)	2.55 (1.93)	Coton de Tulear, Havanese, Min Schnauzer
3	20 (18/2)	3.46 (1.5)	Lhasa Apso, Min Australian Shepherd
4	8 (5/3)	3.21 (1.05)	Rottweiler
5	19 (14/5)	3.19 (1.2)	Dogue de Bordeaux, Pembroke Welsh Corgi, Weimeraner, Wheaton Terrier,
6	11 (10/1)	2.67 (1.37)	Neapolitan Mastiff
7	10 (9/1)	3.36 (1.86)	Bull Mastiff
8	28 (20/8)	2.82 (1.45)	Australian Shepherd, GSD, Golden Retriever, Siberian Husky, Standard Poodle
9	24 (19/5)	2.66 (0.88)	French Bulldog
10	41 (33/8)	3.69 (2.45)	Pomeranian, Shetland Sheepdog, Shiba Inu
11	17 (15/2)	3.68 (1.32)	Bernese Mountain Dog, St. Bernard
12	13 (12/1)	2.73 (1.54)	Italian Greyhound
13	43 (31/12)	3.73 (2.53)	Cairn Terrier, West Highland Terrier
14	17 (14/3)	2.27 (0.95)	Boxer
15	8 (5/3)	3.25 (2.26)	Boston terrier, Cavalier King Charles Spaniel, French Bulldog
16	12 (9/3)	4.45 (1.86)	Maltese, Toy Poodle, Yorkshire Terrier
17	18 (13/5)	3.07 (1.38)	Dachshund, Havanese, Papillion, Pekinese
18	6 (4/2)	5.08 (1.11)	Parsons Russell Terrier, Toy Fox Terrier
19	14 (12/2)	2.92 (0.6)	Boston Terrier, French Bulldog
20	19 (14/5)	3.28 (2.08)	Pomeranian, Shih Tzu
21	12 (8/4)	2.17 (0.59)	Coton de Tulear, Pembroke Welsh Corgi, Shiba Inu, Shih Tzu
22	20 (14/6)	3.21 (1.34)	Havanese, Lhasa Apso, Shih Tzu
23	18 (10/8)	2.16 (0.89)	French Bulldog, Golden Doodle, Golden Retriever, Labrador Retriever, Shiba Inu, Standard Poodle
24	14 (11/3)	3.16 (1.29)	Berna-Doodle, Bernese Mountain Dog, Cavalier King Charles Spaniel, Golden Doodle, Golden Retriever
Total	445 (342/103)	3.15 (1.74)	43

The mean BCS was 3.055 ranging from 2–5.

**Fig 2:**
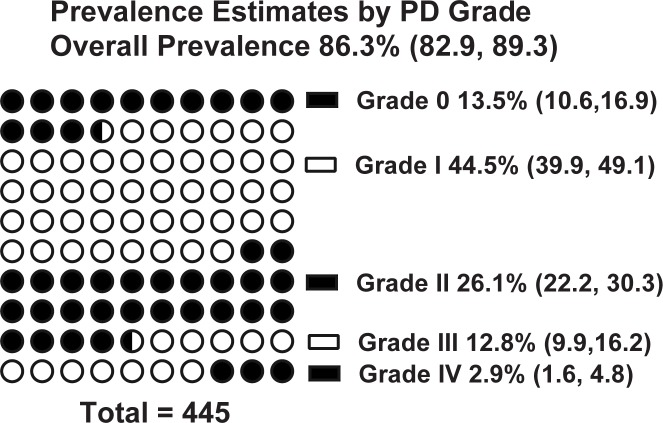
Prevalence estimates. Prevalence estimates (95% CI) by grade of PD.

The overall prevalence of gingivitis or periodontitis (Grades I-IV) was 86.3% (95% CI: 82.9, 89.3) (**Fig 2).**

Age (OR = 1.4; 95% CI 1.24, 1.54; P<0.0001), facility (OR = 1.13; 95% CI 1.09, 1.18; P<0.0001), sex (OR = 1.7; 95% CI 1.12, 2.65; P = 0.013), and NPDS (OR = 2.82; 95% CI 1.34, 5.91; P = 0.006) were found to be associated with a statistically significant increased risk of PD, while provision of a chew item (OR = 0.43; 95% CI 0.24, 0.77; P = 0.005) was found to be associated with a statistically significant decreased risk of PD based upon the results of an ordered logistic regression (OLR) (**Table 2**). All applicable test assumptions were met in this model.

**Table 2:** Results of ordered logistic regression (OLR) NPDS Non-professional dental scaling.

Predictor	OR	95% CI	P
Age in years	1.38	1.24, 1.54	***<0*.*0001***
Breed >36.4 kg	Comparison Group	N/A	N/A
Breed 16.4–36.4 kg	1.96	0.97, 3.97	0.062
Breed 5–16.3 kg	0.97	0.49, 1.93	0.942
Breed < 5 kg	1.89	0.94, 3.69	0.074
Facility	1.14	1.09, 1.18	***<0*.*0001***
Sex	1.73	1.12, 1.53	***0*.*013***
Mesaticephalic	Comparison Group	N/A	N/A
Brachycephalic	1.11	0.72, 1.72	0.632
Dolichocephalic	1.83	0.99, 3.42	0.054
2+ interventions	Comparison Group	N/A	N/A
Chlorhexidine in H2O	1.52	0.76, 3.05	0.238
Chew	0.43	0.24, 0.77	***0*.*005***
NPDS	2.82	1.34, 5.91	***0*.*006***

Of the dogs assessed, 11 breeds (N = 172 dogs) were classified as brachycephalic (Boston Terrier, Boxer, Bullmastiff, Dogue de Bordeaux, French Bulldog, Lhasa Apso, Neapolitan Mastiff, Papillion, Pekinese, Pomeranian, Shih Tzu), 25 breeds (N = 224 dogs) were classified as mesaticephalic (Australian Shepherd, Berna-Doodle, Bernese Mountain Dog, Cairn Terrier, Cavalier King Charles Spaniel, Coton de Tulear, Golden Doodle, Golden Retriever, Havanese, Labrador Retriever, Maltese, Miniature Australian Shepherd, Miniature Pinscher, Miniature Schnauzer, Parson’s Russell terrier, Pembroke Welsh Corgi, Rottweiler, Saint Bernard, Shiba Inu, Siberian Husky, Toy Fox Terrier, Weimaraner, Wheaton Terrier, West Highland Terrier, Yorkshire Terrier,), and 6 breeds (N = 49 dogs) were classified as dolichocephalic (Dachshund, German Shepherd Dog, Italian Greyhound, Shetland Sheepdog, Standard Poodle, Toy Poodle). No association between skull morphology and PD severity was identified **(Table 2).**

Analysis of IRR found percent agreement between the two raters to be 86.2%, the weighted kappa was 0.4731 with 95% CI 0.3847, 0.5615 indicating moderate agreement [23].

All owners provided some type of preventive care to their dogs, most commonly chlorhexidine in the water, some type of chew item, NPDS, or a combination of two or more of these interventions. Additionally, all dogs were fed a commercially available dry dog food and had a dental examination performed by a veterinarian at least once a year.

## Discussion

This study has several important findings. First, no association between BCS and severity of PD was found likely due to the lack in variability of BCS; the majority of dogs were in ideal body condition. Secondly, the prevalence of gingivitis or PD in this population (Grades I-IV) was 86.3%. Wiggs and Lobprise [2] have reported a prevalence of 80% in companion dogs 2 years of age or older while others have reported lower prevalence levels. For example, in a cross-sectional study of 31,484 dogs examined at 52 private veterinary practices, Lund et al. [24] found a prevalence of 20.5% for dental calculus and 19.5% for gingivitis. More recently, an analysis of electronic veterinary records from a random sample of 3,884 (out of 148,741) dogs from 89 clinics in England reported a prevalence of PD of 9.3% (95% CI: 8.3–10.3) [3]. Further, a study of 408 dogs presenting to private clinics in the Czech Republic reported a 60.8% prevalence of periodontitis, 61.3% of calculus, and missing teeth in 33.8% of dogs examined [5]. Possible explanations for the differences in reported prevalence include inconsistent coding and case definitions. For instance, the study by Lund et al. [24] included any degree of PD whereas the study by O’Neill et al. [3] only included conditions that required immediate treatment as determined by the attending veterinarian. Alternatively, populations in different parts of the world may in fact have different prevalence levels of PD. In future studies, consistent definitions of PD, assessment tools being used, and reporting of IRR should be considered for a more accurate comparison of studies.

In the current study, of the 86.3%% of dogs with PD, 45% were assigned a grade of I, meaning the dog had gingivitis. Gingivitis is an inflammation of the gums caused by the accumulation of plaque on the teeth. At this stage, veterinary recommendations are typically dental prophylaxis or active homecare (e.g. brushing) to reverse or halt the progression to periodontitis. However, not all cases of gingivitis progress to periodontitis and so it has been suggested that including grade I in prevalence estimates of PD may overstate the problem as gingivitis is of little clinical importance except as a marker for oral hygiene [25]. In this population gingivitis may be an important marker in assessing the effectiveness of the preventive care being utilized and as a means of identifying individuals in need of closer monitoring for progression of PD.

As previously reported in companion dogs [5], [7], increasing age was associated with increased risk of PD and while the OR was 1.86 for dogs < 5 kg (i.e. small breed size), it was not statistically significant. Kyllar and Witter [5] reported higher prevalence of PD, dental calculus and missing teeth in breeds that were less than 10 kg compared to breeds that were 10–30 kg or greater than 30 kg in all age categories (1–4 yrs, 5–8 yrs, 9–11 yrs, 12–13 yrs). Harvey et al.[7] examined 1,350 dogs under anesthesia in veterinary hospitals in North America and reported that calculus, periodontal (gingival) inflammation, and periodontal attachment loss were common in companion dogs and increased in severity with increasing age while decreasing in severity with increasing body weight. Additional studies conducted from 1968–1987 reviewed in Harvey et al. [7] also reported an association between increasing prevalence of PD and increasing age (N = 7 studies) and decreasing prevalence with increasing body weight (N = 4 studies).

While the prevalence of PD was similar in this population to that reported in companion dogs, increased risk of systemic disease associated with PD is still a matter for concern. Physiologic parameters that have been reported to positively correlate with gingivitis include globulin concentrations, platelet count, microalbuminuria, ALP and ALT activity while creatinine concentration was negatively correlated [26]. Additionally, several variables have been shown to be markedly reduced after prophylactic cleaning including serum C-reactive protein (CRP) (a marker of inflammation), albumin and BUN concentration, urine specific gravity (USG), and blood glucose concentration [26] leading the authors to conclude that there was an association between severity of PD and systemic inflammation. Whyte et al. [27] found a significant positive correlation between ALT and PD and a negative correlation between platelet count and PD. In addition to renal, hepatic and systemic inflammation, it has been suggested that PD may be associated [28, 29] with increased risk for cardiovascular problems such as endocarditis and cardiomyopathy. For example, Glickman et al. [28] reported that dogs with PD had a higher frequency of cardiovascular-related events and that the frequency increased with increasing severity of PD. Others have questioned the validity of these results indicating more research is needed before definitive conclusions can be drawn [29]. These studies suggest that any degree of PD increases risk for several systemic diseases and merits concern. Future studies should aim to characterize the degree of systemic disease in this population as well as the impact on pregnancy outcomes.

We hypothesized that brachycephalic breeds would be at an increased risk of PD compared to mesaticephalic and dolichocephalic breeds. Brachycephalic breeds are predisposed to malocclusions associated with mandibular mesioclusion as well as overcrowding and misalignment of teeth. Problems associated with malocclusion include difficulty chewing, abnormalities in temporomandibular joint (TMJ) function, soft tissue trauma, and premature tooth loss caused by PD due to crowding and rotation of the teeth [30], [31]. The results of this study did not identify skull morphology as a risk factor for PD. One explanation is that breed size was a confounding factor. Future research should aim to assess the impact of skull morphology on PD in breeds of similar sizes.

Another important finding is that all of the facility owners in this study provided some type of preventive care, most commonly the addition of chlorhexidine solution to the water system, provision of chew items such as cow hooves, or NPDS. None of the facilities practiced routine brushing of their dogs’ teeth. All dogs had a dental examination by a veterinarian at least yearly. It is encouraging to find that facility owners were aware of the importance of preventive dental care and were providing some form of intervention. To our knowledge, this is the first study to try and assess the effectiveness of these interventions. Nine facilities reported performing NPDS, scraping the teeth with a dental instrument to remove calculus, at least once a year. Aside from the risk of pain and injury to the dog from this procedure, it is ineffective at removing subgingival plaque and bacteria. Westfelt et al. [32] reported that supragingival plaque control failed to prevent the progression of PD over a period of 3 years in patients with advanced PD. The American Veterinary Dental College (AVDC) is opposed to the practice for these reasons as stated on their website http://avdc.org/AFD/category/facts/. In this population, dogs that underwent this procedure were at increased risk of PD (OR = 2.82) in support of the AVDC’s position.

Eleven facilities added chlorhexidine solution to the water system. Several studies have shown the effectiveness of chlorhexidine in decreasing inflammation and gingivitis. It has been shown to be safe, has little systemic uptake, and no known bacterial resistance has been documented [33]. Optimally, the gel or solution should be applied directly on the teeth and gums of the dog [33], [34]. While this seems like a useful intervention, its effectiveness when added to the water supply has not been documented and in this study there seemed to be no beneficial effect (OR = 1.52; 95% CI 0.758, 3.053; P = 0.238). In this study, facility management differed in the concentration used as well as the schedule of use which may have impacted the results. Evaluating the efficacy of chlorhexidine water additives in promoting dental health should be a goal of future research in preventive care.

Twelve facilities provided some sort of chew item, most commonly cow hooves. The effectiveness of several dental chews has been documented by the Veterinary Oral Health Council (VOHC) [35] but cow hooves are not among them. Chew items are generally most beneficial in removal of supragingival plaque and calculus on the cheek teeth [36], [37]. One concern with any chew item is the risk of tooth fracture, which should be a factor in dental health management decisions. In this population, provision of a chew item seemed to have a protective effect on PD (OR = 0.43; 95% CI 0.24, 0.77; P = 0.005). Future research is needed to evaluate the effectiveness of the most commonly used chew items in CB facilities.

All dogs were fed a commercially available dry kibble which should ensure that they received the proper nutrition to maintain periodontal health. It has been conventional wisdom that a dry food diet is better for oral health than a wet or canned food diet, but this view has been challenged [8]. Only veterinary dental diets have been documented to be effective as a dietary preventive oral health measure [33], [38]. In the population of dogs housed at CB facilities, cost may be a limiting factor that constrains feeding such diets, but more importantly, dental diets do not meet AAFCO standards for all life stages. Therefore it is not advisable for a breeding kennel to attempt to maintain pregnant and lactating bitches on dental diets [39], [40].

IRR is the degree to which different raters draw the same conclusions when evaluating the same subject [41]. In this study, IRR was evaluated using both percent agreement and a weighted kappa statistic. Kappa statistics take into account the effect of agreement due to chance in a manner that percent agreement cannot. A kappa value of 0 indicates that the agreement between raters is no greater than expected due to chance. Weighted kappa allows the degree of disagreement between levels in an ordinal scale to be accounted for (e.g. the disagreement between 0 and 3 is greater than that between 1 and 2) [23]. The moderate agreement identified between the observers in this study indicates that there is room for improvement in rater training for the use of this subjective scale. Both of the raters had experience in the veterinary clinical setting and could be expected to be more likely to agree than individuals with less practical experience. Nevertheless, future studies should aim to include raters with less experience.

This study did not take into account the validity of the raters’ assessments. The reference standard for diagnosing PD in dogs is a full oral examination, prophylactic cleaning, and dental radiographs performed with the dog under anesthesia. These types of assessments were not feasible in this study, but detection of PD can have implications for the welfare of an individual dog or population of dogs. Therefore developing a valid visual assessment tool for use in the field is important. Future research should aim to validate the visual use of the scale with scoring during dental prophylaxis.

In addition to the need for validation of the visual scoring against radiographs, this study had further limitations. First, breed size standards were used as a surrogate for the body weights of individual dogs. In future, weighing each dog during examination would be ideal. All dogs were from the same geographic area of the country and all facilities were owned by members of the Amish community. Future studies should aim to assess populations in other areas of the country using more diverse management practices. Additionally, most breeds were located at only one or two facilities, limiting the ability to differentiate breed effect from facility or management effects on PD prevalence and severity. Finally, selection bias cannot be ruled out since all participants volunteered for the study.

Despite the study’s limitations, our findings represent an important first step in objectively characterizing dog dental health in CB kennels, as to our knowledge, this is the first reported prevalence estimate of PD in such dogs. Our results follow trends previously reported in companion dogs suggesting that in CB kennels, where owners make an attempt to address dental health, this population may not have more severe disease than companion dogs in homes. As with caretakers of companion dogs, more education about the importance of appropriate preventive dental care is needed. Most importantly, development of a cost effective, easy to use intervention to improve compliance is necessary to prevent PD in CB kennels, as is the case for all dog populations.

## Supporting information

S1 Appendix Data set. (XLSX)
